# Physiological responses of gopher tortoises (*Gopherus polyphemus*) to trapping

**DOI:** 10.1093/conphys/coab003

**Published:** 2021-02-13

**Authors:** Jeffrey M Goessling, Mary T Mendonça

**Affiliations:** 1 Department of Biological Sciences, Auburn University, AL 36849 USA; 2 Natural Sciences Collegium, Eckerd College, 4200 54^th^ Ave S, St Petersburg FL 33711 USA

**Keywords:** Ecoimmunology, heterophil:lymphocyte ratio, lactate, stress

## Abstract

With a growing number of species of conservation concern, understanding the physiological effects of routine sampling of vertebrate species remains a priority to maintain the welfare status of wildlife and ensure such activities are not counter to conservation goals. The gopher tortoise (*Gopherus polyphemus*) is a species of conservation concern throughout its range and is among the most frequently trapped turtles globally (for both research and conservation activities). Several studies have found equivocal results on the effects of trapping and handling on the glucocorticoid stress response. In this study, we tested how multiple physiological biomarkers (i.e. plasma lactate, corticosterone (cort), heterophil:lymphocyte ratio (HLR) and bactericidal ability (BA)) respond to four different combinations of trapping conditions in comparison to baseline reference sampling. We found that trapping and handling of gopher tortoises yielded a rapid rise in plasma lactate concentration followed by elevations of cort and stress-associated immune changes. In visibly distressed animals that were in traps for fewer than 2 hours, lactate, cort, HLR and BA were all elevated, and generally more so than animals that remained calm in traps for a similar amount of time. Animals that had been trapped and then held for a 3-hour restraint showed similar degrees of physiological alteration as those that showed outward signs of distress. This study demonstrates that trapping may yield physiological disturbances in gopher tortoises, although the intensity of this response is highly variable between individuals and the duration of such alterations remains unknown. This research emphasizes the need for continued work to refine trapping and handling processes in an effort to minimize impacts on individuals and populations.

## Introduction

A common approach to assessing how wildlife respond to natural and anthropogenic challenges is to quantify various physiological biomarkers related to stress, most notably by assaying baseline glucocorticoid concentrations (cort) (Romero and Reed, 2005). Thus, physiological biomarkers are used to quantify environmental or allostatic loads (McEwen and Wingfield, 2003) on individuals and are inferred as a comparison of stressors among populations, which has direct conservation and management implications. However, physiological biomarkers themselves do not directly measure burdens on individuals, but rather they quantify specific physiological responses to potential or real burdens (MacDougall-Shackleton *et al.,* 2019). One result of this implicit gap between stressors and their expected biomarkers is that biomarkers, especially cort, do not always predictably indicate hypothesized stressful scenarios on vertebrates (Goessling *et al.,* 2015; MacDougall-Shackleton *et al.,* 2019). As a result, ecologists have sought alternative methods to quantify vertebrate stress by measuring a suite of physiological responses to environmental conditions.

As downstream effects of glucocorticoid stress responses, the immune system changes in predictable ways that act as positive indicators of both acute (i.e. short term) and chronic (i.e. long term) stressors. Specifically, the heterophil (or neutrophil): lymphocyte ratio (HLR) increases in response to acute stress and remains elevated throughout chronic stress exposure (Davis *et al.,* 2008; Goessling *et al.,* 2015). The elevation in HLR not only positively indicates environmental stress, but it also does not attenuate as rapidly as glucocorticoid concentration in response to prolonged stress. This change in HLR is considered an adaptive response that prepares the immune system for short-term survival (Davis *et al.,* 2008). Other changes in immunity have been noted in acutely stressed animals that can be further considered adaptive for short-term survival, such as increases in bactericidal ability (BA) during restraint (Hopkins and Durant, 2011).

During periods of increased metabolic demand, and at the onset of oxygen debt (such as fleeing from predators), vertebrates transition from aerobic to anaerobic metabolism, which initiates a rise in plasma lactic acid concentration (Feder and Arnold, 1982; Hawthorne and Goessling, 2020). The rise in lactic acid can be so severe that it can lead to immobilization and even death (Seymour *et al.,* 1987). Therefore, if an initial signal driving high metabolic demand (e.g. predation avoidance) may not be a physiological stressor itself, an altered and stress-associated physiological state may result from a rise in lactic acid. In chickens exposed to a restraint stress, plasma lactate increases to its maximum peak in less than a minute following restraint, while cort gradually increases to a maximum concentration 7 minutes following the initiation of restraint (Voslarova *et al.,* 2011). Thus, one interpretation of the response to restraint includes a physiological change (e.g. rising [lactate]) that could stimulate subsequent glucocorticoid-associated stress responses. Because non-avian reptiles have low respiratory efficiency compared to endotherms, they may be more prone to negative effects of lactic acid build up under natural conditions in response to either pursuit or restraint. Thus, responses to the buildup of this anaerobic waste product are likely an ecologically relevant pressure that has shaped physiological stress responses. Kemp’s Ridley sea turtles experience lactic acidosis when trapped in fisheries trawls (Stabenau *et al.,* 1991) and there are numerous known effects of lactic acidosis on crocodilians subjected to manual restraint (Franklin *et al.,* 2003). Among turtles, most research has examined lactic acidosis tolerance in submerged anoxic aquatic turtles (e.g. Jackson, 2000). Among terrestrial turtles, Goessling *et al.* (2018) found that gopher tortoises (*Gopherus polyphemus*) alter metabolic rates during dormancy, and may even become partly anaerobic under colder conditions.

Understanding how to best quantify stress, and if and how handling alters the physiology of sampled individuals is vital to studying imperiled species. In actively managed species that are regularly handled, understanding the specific handling effects on physiological systems is crucial to developing effective conservation practices that minimize negative effects of stress on populations. Gopher tortoises are of conservation concern throughout their range and have suffered severe human-associated declines (Auffenberg and Franz, 1982). Because of their high site fidelity, slow growth rates and high longevity, gopher tortoises are also intensively managed with translocation (Tuberville *et al.,* 2008; McCoy and Berry, 2008; Cozad *et al.,* 2020) which often requires trapping and/or prolonged handling. For example, more than 5000 gopher tortoises have been translocated into one site in Florida from across that state (Cozad *et al.,* 2020). Between 2009 and 2019 in Florida, 52 613 gopher tortoises were permitted for translocation (Gopher Tortoise Candidate Conservation Agreement, 2020). Both ethical and practical questions remain for conservation practitioners using translocation to manage this species (McCoy and Berry, 2008), and understanding the direct effects of trapping and handling on individual tortoises is a first step to assess this broader conservation tactic. We are aware of two studies that have addressed how trapping (Ott *et al.,* 2000) and handling (Kahn *et al.,* 2007) affect stress in gopher tortoises, as measured via cort, and both of these studies were equivocal as to how deleterious these activities are to tortoises. Ott *et al.* (2000) found that cort did not significantly increase in response to normal trapping activities and that prolonged time (i.e. 12 hours) in a trap only resulted in slight cort increase; Kahn *et al.* (2007) found that there was no effect of handling on cort. To that end, the objective of this study was to reexamine the physiological effects of trapping on gopher tortoises using additional physiological biomarkers (e.g. [lactate], HLR and BA).

## Materials and methods

To assess the effects of trapping on physiological parameters, we followed a strict sampling protocol for both trapped and untrapped animals.

All blood samples were collected from tortoises within 3 minutes of initial human contact in sterile pre-haparinized syringes with a 25-gauge needle affixed to the syringe (Goessling *et al.,* 2016). Blood was collected from the sub-carapacial sinus. Whole blood was stored on ice for no longer than 2 hours, following which the plasma fraction was isolated via centrifugation for ~ 8 minutes at 8000 RPM and stored in liquid nitrogen. While in the field, a blood smear was made, methanol fixed and later stained with a Hema 3 differential stain kit according to manufacturer’s instructions (Fisher Scientific). Differential leukocyte counts were made by counting 100 total leukocytes on each slide according to Goessling *et al.* (2016). Heterophil: lymphocyte ratios (HLR) were subsequently calculated from differential leukocyte counts.

Plasma BA assays were performed using a standard plating technique as described in Goessling *et al.* (2016). To measure cort, 30 ul of plasma were extracted in anhydrous diethyl ether, resuspended in assay buffer and assayed on an Enzo corticosterone Enzyme Immunoassay (EIA) (Cat no. ADI-900-097). Because of a wide range of CORT concentrations, samples were considered to be equal to 1 ng/ml if they were below this reliable threshold for our samples on the EIA kit. Plasma lactate concentration was measured using a Nova ® Lactate Plus Meter (Hawthorne and Goessling, 2020).

We collected blood samples from animals exposed to one of five different handling/trapping conditions, which included combinations of baseline and stressor-induced sampling. We treated three of the five sub groups as ‘knowns’ and were as follows: hand captured baseline sample, referred to as ‘known-untrapped baseline’; trapped with no visible signs of distress and in trap less than 2 hours, referred to as ‘duration known-nondistressed’; and in trap for less than 2 hours with visible signs of distress, referred to as ‘duration known-distressed’. The trapping methodology included surveyors checking traps twice daily (between 1000–1200 h and 1400–1600 h), thus for the known-duration animals, we confirmed that a tortoise was not in the trap for longer than 2 h prior to sampling the animal; the ‘unknown’ animals were detected in a trap more than 2 hours and up to 18 hours since the last trap check, thus we could not ascertain a precise estimate of time in trap. For these unknown time-in-trap animals, we either collected a blood sample immediately upon detection of the tortoise (referred to as ‘unknown’) or we temporarily stored the tortoise in a plastic bin for 180 minutes prior to blood collection, thus adding a restraint challenge (referred to as ‘unknown-restrained’). Each of these five conditions was necessary for us to effectively address our research questions. The known-untrapped baseline blood sample provided a standard minimum reference level for subsequent stressful conditions. The known-distressed category included animals that had foamy saliva on their forearms and/or face due to high respiratory demand and/or had some mild abrasions associated with rubbing against the trap. In all conditions, we attempted to minimize all stress and discomfort experienced by tortoises by providing shade over traps. All animals that had been in the trap less than 2 hours served as a treatment group that may represent a ‘best case scenario’ for research and management activities. The ‘unknown’ categories were included in this study because these samples represented a realistic scenario for tortoises involved in intensive management activities, such as trapping for translocation and mitigation. Additionally, animals in the unknown groups did not display external signs of stress as were seen in the distressed group above. The unknown-restrained group was included in this study as a comparison for normal research and management practices where tortoises may be processed, but then are handled/restrained for a short period of time to move, mark, or collect additional data from the animal.

All statistical analyses were conducted in SigmaPlot 14.0 (Systat Software Inc). To normalize data, natural log-transformed BA and lactate concentration were compared between the five conditions using a one-way ANOVA; cort and HLR were compared between the five conditions using a Kruskal–Wallis one-way ANOVA on ranks with a Dunn’s multiple comparison post-hoc test to identify where significant differences were present. Additionally, to examine direct relationships among some of the physiological parameters, linear regressions were performed between ln-transformed lactate and ln-transformed cort and between ln-transformed BA and ln-transformed cort.

One final analysis, a regression between HLR and cort, was conducted using parameters from the three known categories to test whether these two frequently utilized ‘stress biomarkers’ could similarly and consistently identify individual tortoises that were distributed between three known and visibly apparent statuses of stress (e.g. true baseline, calm in a trap and visibly distressed in a trap).

## Results

A total of 68 individual tortoises were sampled in this study, which included 20 ‘knowns’ comprised of the following: 7 known-untrapped baseline, 6 duration known-nondistressed and 7 duration known-distressed samples. A total of 40 unknown and 8 unknown-restrained samples were collected.

In general, data on all potential stress parameters were highly variable between individuals, yet a pattern was present that revealed visibly distressed individuals are physiologically stressed, and that among all of the stress categories, the stress parameters are positively related to each other.

**Figure 1:**
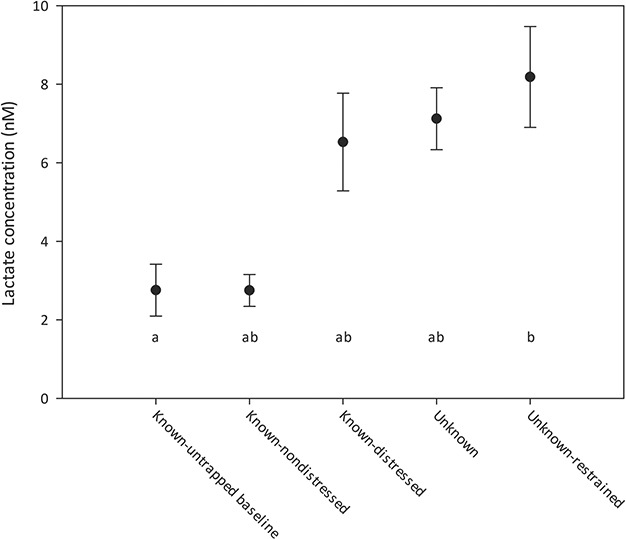
Gopher tortoises were sampled for lactate across five conditions (see text for full descriptions). Points indicate mean values and bars indicate standard error. Significant groupings are indicated by letter assignments.

Plasma lactate concentration ranged from 0.8 to 16.1 mM, and the average across all individuals was 6.4 mM. The lowest average lactate concentration was in the hand-captured tortoises at 2.8 mM and the highest mean lactate concentration was in the unknown-restrained group at 8.2 mM; these two groups were also significantly different than each other, with the other three groups intermediate (Fig. 1; *F* = 3.42, *P* = 0.014).

**Figure 2:**
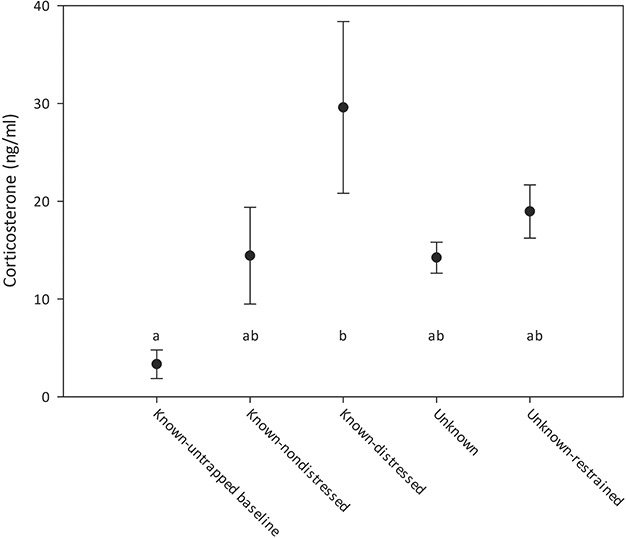
Gopher tortoises were sampled for corticosterone across five conditions (see text for full descriptions). Points indicate mean values and bars indicate standard error. Significant groupings are indicated by letter assignments.

Plasma cort was highly variable among individuals and groups. The most variable group was the known-distressed tortoises, which ranged in cort from 4.5 ng/ml to 55.8 ng/ml, and averaged 23.2 ng/ml. The known trapped-distressed group had the highest mean cort among the groups and was significantly higher than the hand-captured tortoises, which also had the lowest average cort equal to 3.3 and included three samples below the 1.0 ng/ml assay threshold (*H* = 14.85, *P* = 0.005; Fig. 2).

**Figure 3:**
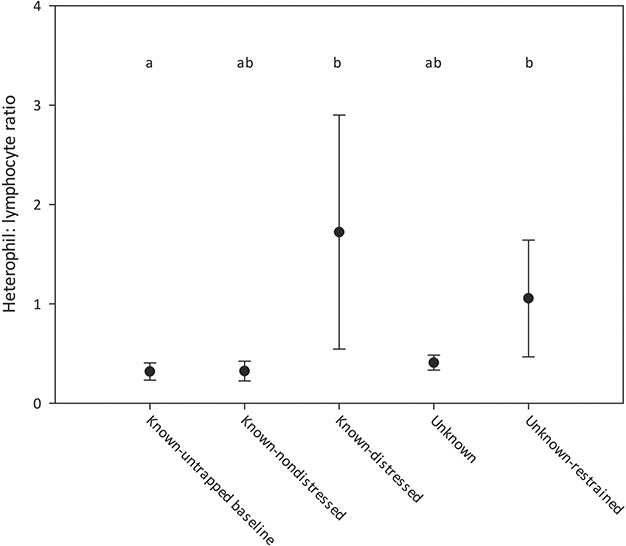
Gopher tortoises were sampled for heterophil:lymphocyte ratio across five conditions (see text for full descriptions). Points indicate mean values and bars indicate standard error. Significant groupings are indicated by letter assignments.

HLR were significantly elevated in both the visibly distressed tortoises as well as the unknown-restrained tortoises in comparison to the known-baseline tortoises (*H* = 15.08, *P* = 0.0005; Fig. 3). While not statistically significant, the duration-known nondistressed and unknown tortoises had similarly low mean HLR as the hand-captured tortoises. Additionally, the only groups with HLR that might be considered ‘low’ (i.e. HLR < 1.0, Goessling *et al.,* 2016) were the known-untrapped baseline, duration known-nondistressed and unknown, while the tortoises with ‘high’ mean HLR were both the duration known-distressed and the unknown-restrained tortoises.

**Figure 4:**
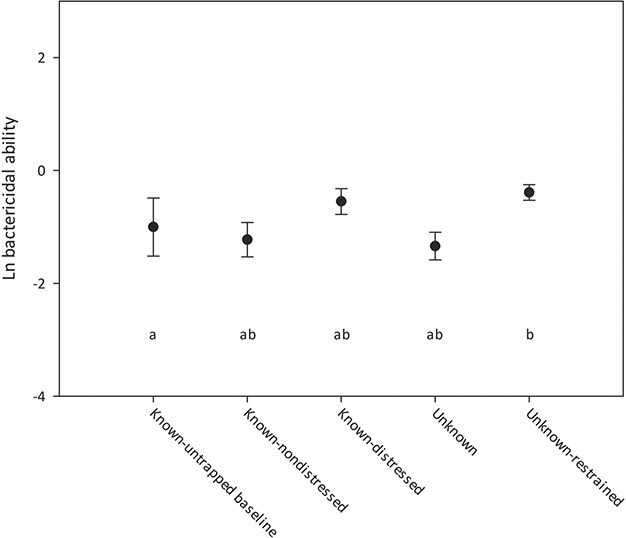
Gopher tortoises were sampled for bactericidal ability across five conditions (see text for full descriptions). Points indicate mean values and bars indicate standard error. Significant groupings are indicated by letter assignments.

BA was only significantly elevated in the unknown-restrained tortoises compared to the known-untrapped baseline (*F* = 3.24, *P* = 0.018; Fig. 4). The second-highest mean BA was in the duration known-distressed tortoises, which was not significantly different than the other sample means.

**Figure 5:**
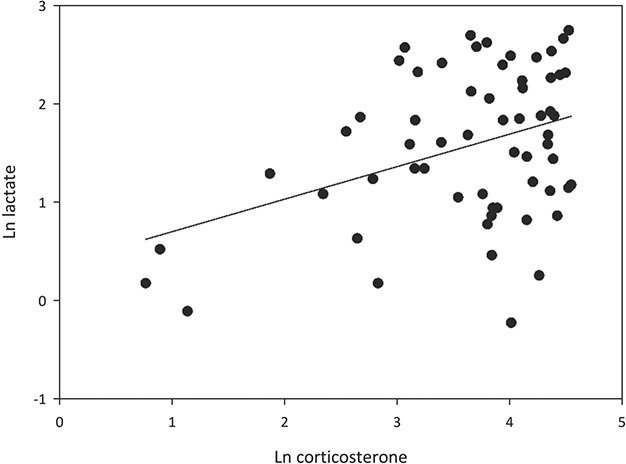
A significant positive relationship was found between lactate and corticosterone (linear regression, *T* = 2.640, *P* = 0.011, *R*^2^ = 0.107).

**Figure 6:**
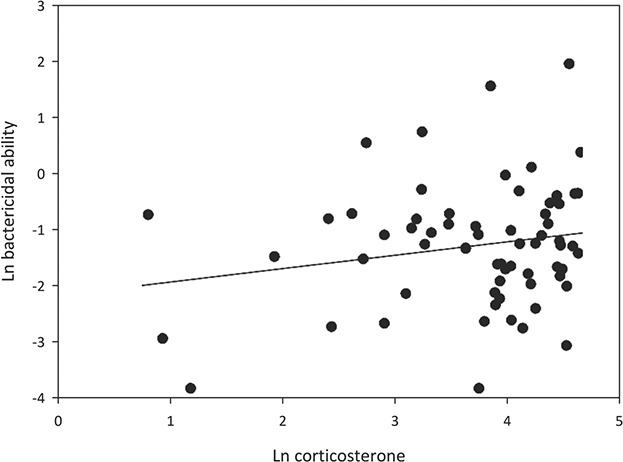
A significant positive relationship was found between bactericidal ability and corticosterone (linear regression, *T* = 7.187, *P* < 0.001, *R*^2^ = 0.115).

**Figure 7:**
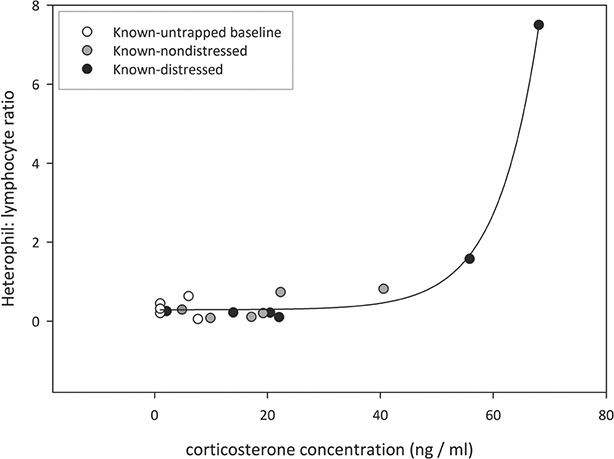
A positive relationship was found between heterophil: lymphocyte ratio and corticosterone in ‘known’ tortoises in each of the three groups of untrapped baseline (white), duration known-nondistressed (gray) and duration known-distressed (black) (three-parameter nonlinear regression, *t* = 10.147, *P* < 0.0001, *R*^2^ = 0.986).

Corticosterone concentration was significantly positively related to each of the parameters measured in the study (Figs 5–7). Most strikingly, the exponential positive fit (Fig. 7, *P* < 0.0001) between HLR and cort in the three groups of ‘known’ suggests that HLR generally is slow to respond to an increase in glucocorticoid, up to a certain level at which point HLR rapidly rises. Also of note in this relationship is how the three groups are spaced along the cort axis, with hand-captured tortoises the lowest, trapped tortoises the intermediate and visibly distressed the highest.

## Discussion

As species are increasingly faced with demographic instability, the need to capture animals for research and management activities is becoming more critical; this is especially the case for reptiles in general (Gibbons *et al.,* 2000) and turtles specifically (Lovich *et al.,* 2018). This study demonstrated that trapping, in general, is a relatively minimal stress to most gopher tortoises, as evidenced by the unknown individuals, which had low lactate, cort, HLR and BA. Tortoises in that category had been in a trap for between 2 and 18 hours and generally appeared normal and non-distressed. While not significantly different, the unknown-trapped tortoises tended to have higher mean lactate concentration, which grouped more closely with the visibly distressed tortoises. Because lactate was highly variable among and within groups, this pattern was not statistically significant, although this likely provides an indication about the progression of the tortoise physiological response to trapping. First, this parameter was quite variable, which indicates not all individuals respond to trapping in the same manner. The tortoises that move more upon trapping likely do so at the cost of a surge in lactate, which likely initiates a glucocorticoid stress response (Voslarova *et al.,* 2011). Because not all individuals respond by attempting to escape from the trap (some remained quiescent in the trap and move infrequently, pers. observ.), it would be predicted that not all individuals will display the same lactate surge in response to trapping. While BA is not typically considered a stress biomarker, Hopkins and Durant (2011) found that this innate immune parameter increased in response to handling time. Similarly, in this study, we found that tortoises exposed to an additional handling stress, specifically those held for a 3-hour restraint period after a longer than 2 hour period in a trap (i.e. unknown-restraint) exhibited significantly higher BA than the hand-captured tortoises. Taking into account the known stress-associated increases in HLR, both of these immuneparameters likely prime individuals to respond to costly and acutely stressful and/or physically traumatic scenarios. Such events might demand an up-regulation of innate immunity to initiate healing following traumatic injury. It is therefore conceivable that using BA as another biomarker of stress could provide greater perspectives in a generally under-studied link between immunity and stress in wild populations. This relationship between stress and increases in BA is also supported by the significant positive relationship that we detected between cort and BA (Fig. 6).

Tortoises that had shown visible signs of distress (e.g. panting, skin abrasions, etc.) and that were in traps for approximately 2 hours or less had relatively high lactate, the highest mean cort concentration, the highest mean HLR and the highest mean BA (although this mean was not significantly different than the other groups). Together these parameters should be interpreted as a clear marker of a physiological stress response, consistent with the visible observations of distress. While the pattern was not well resolved in their study, Ott *et al.* (2000) found that longer durations in trap trended to display increased cort in this species. Comparing our data to previous studies of gopher tortoise trapping stress clearly indicate that individuals are highly variable in how they respond behaviorally and physiologically to trapping.

Our data demonstrate that certain aspects of trapping, and more importantly, that individual tortoise responses to trapping, can initiate a sequence of stress responses. In both the known-trapped and known-distressed tortoises in this study, the only difference between these groups was the individual tortoises’ responses to trapping. Because this study was conducted at the same sites and same seasons, individual tortoise personality is likely a strong determinant of the physiological response to trapping. As noted above, upon capture, some gopher tortoises will sit quietly in the back of a trap, while others struggle in the traps and thus initiate the noted stress responses. Across our years of sampling gopher tortoises for mark-recapture demographic studies (e.g. Goessling *et al.,* 2021), we have not observed a strong pattern indicating that prior tortoise trapping experience determines the behavioral responses to trapping; rather, this response appears to uniquely vary by individual. This pattern is likely one that applies more broadly across vertebrate populations, as more evidence reveals previously undocumented personality differences in behavior among individuals. It is thus likely that certain individuals can be expected to respond with stress, whereas others are not likely to do so. This difference in response to trapping might be consistent with the shyness-boldness behavioral traits in lizards (Waters *et al.,* 2017). Follow up research should consider how the HPA axis may either respond to or be the cause of personality differences in individual responses.

This study was designed in a way as to understand how a potential external stressor (e.g. trapping/handling) drives an internal stressor (lactate), which might subsequently initiate a glucocorticoid response with associated changes in immunity. In general, the data supported that the progression of the stress response was associated with trapping efforts and causes a range of physiological responses, depending on where each individual was along the stress axis. A series of future experiments that control a combination of lactate and glucocorticoid could establish causality between endogenous physiological stressors and the glucorticoid response to such stressors. Additionally, because the glucocorticoid response promotes high metabolic performance, it is likely that the lactic acid-glucorticoid relationship may prepare individuals for future aerobically demanding functions (Lipowska *et al.,* 2019). At baseline, the hand-captured tortoises had the lowest means of all physiological parameters. The tortoises that were calm and in traps for less than 2 hours had similarly low levels of most parameters, while the tortoises that were distressed and in traps for less than 2 hours had already initiated a rise in lactate, likely associated with heightened physical activity of trying to escape from a trap. As was also predicted, tortoises that had been exposed to a known stressor (i.e. handling for 3 hours following trapping), had high lactate, cort, HLR and BA.The progression of the physiological responses to captivity (e.g. rise in lactate) in trapped tortoises drives the more downstream stress effects of an increase in plasma glucocorticoid and eventual immune effects.

An interpretation of a model of the stress response in ectotherms applied to our study thus follows from the upstream cause (i.e. trapping) to numerous sequential downstream stress responses. These changes follow: (i) differences in behavioral responses to captivity in a trap leads to (ii) increased metabolic demand causing a lactic acid rise, (iii) corticosteroid increases in response to the stressor of trapping and (iv) an upregulation of innate components (e.g. HLR and BA) of the immune response. While understanding the cause and progression of stress responses in taxa of conservation concern is of great value, a large data gap exists in understanding the duration of such acute responses to stress. Specifically, determining what length of a stressful experience causes increased or attenuated stress responses (Fazio*et al.,* 2014; Goessling *et al.,* 2015) will determine what type of population sampling might run counter to conservation goals for populations that are subjects of intense research or management activities (McMahon *et al.,* 2012; Lindsjö *et al.,* 2016). For example, the trapped-unknown tortoises in this study tended to have slightly elevated lactate, but were low for all other measures. Determining the amount of time in a trap that transitions animal from this mild response to the more intense glucocorticoid response seen in the restrained tortoises might offset potentially negative and stressful effects of trapping on wildlife. Additionally, knowing the duration of time that glucorticoid remains elevated following a single stressful experience is key to assessing the risk to wild populations.
